# MicroRNA-21 promotes proliferation, migration, and invasion of colorectal cancer, and tumor growth associated with down-regulation of sec23a expression

**DOI:** 10.1186/s12885-016-2628-z

**Published:** 2016-08-05

**Authors:** Chenli Li, Lingxu Zhao, Yuan Chen, Tiantian He, Xiaowan Chen, Jiating Mao, Chunmei Li, Jianxin Lyu, Qing H. Meng

**Affiliations:** 1 Key Laboratory of Laboratory Medicine, Ministry of Education of China, Zhejiang Provincial Key Laboratory of Medical Genetics, School of Laboratory Medicine and Life Sciences, Wenzhou Medical University, Wenzhou, Zhejiang 325035 China; 2 Department of Laboratory Medicine, The University of Texas MD Anderson Cancer Center, Houston, TX 77030 USA

**Keywords:** Colorectal cancer, miR-21, Sec23A, Proliferation, Tumor growth

## Abstract

**Background:**

MicroRNA-21 (miR-21) is up-regulated in many cancers, including colorectal cancer (CRC). Nevertheless, the function of miR-21 in CRC and the mechanism underlying that function is still unclear.

**Methods:**

After analyzing the expression of miR-21 and Sec23A in CRC cell lines, we transfected the highest miR-21 expressing cell line, SW-480, with a plasmid containing an miR-21 inhibitor and the lowest miR-21 expressing cell line, DLD-1, with a plasmid containing an miR-21 mimic and measured the effects on the expression of Sec23A and on cell proliferation, migration, and invasion. We also evaluated the effect of knocking down Sec23A on miR-21 expression and its effects on cell proliferation, migration, and invasion. Finally, we assessed the effect of miR-21 in a xenograft tumor model in mice. Tumor tissues from these mice were subjected to immunohistochemical staining to detect the expression of Sec23A.

**Results:**

Genetic deletion of miR-21 suppressed the proliferation, migration, and invasion of SW-480 cells, while over-expression of miR-21 promoted proliferation, migration, and invasion of DLD-1 cells. Inhibition of miR-21 increased the expression of Sec23A protein in SW-480 cells while over-expression of miR-21 significantly suppressed the expression of Sec23A protein and Sec23A mRNA in DLD-1 cells. Knockdown of Sec23A increased the expression of miR-21 in SW480 and DLD-1 cells and their proliferation (DLD-1 only), migration, and invasion. Over-expression of miR-21 promoted tumor growth in BALB/c nude mice and suppressed tumor expression of Sec23A.

**Conclusion:**

These findings provide novel insight into the molecular functions of miR-21 in CRC, which may serve as a potential interesting target.

## Background

Colorectal cancer (CRC) is the third most common cancer and the fourth most common cause of cancer-related death worldwide [1]. While chemotherapy is usually effective in reducing tumor cell growth and counteracting metastatic progression [2], it often loses efficacy, in advanced CRC through development of chemoresistance [3, 4], leading to disease recurrence and often patient death. Thus, seeking for new therapeutic approaches are needed to overcome this resistance, and targeted therapies are believed to offer the greatest promise.

MicroRNAs (miRNAs) belong to a class of small endogenous RNAs that influence many biological processes through binding to the 3′- untranslated region of target messenger RNA (mRNA), mediating either mRNA degradation or translational repression [5]. Aberrant miRNA expression is associated with many diseases, including cancers [6–8]. Accumulating evidence indicates that miR-21 is involved in the pathogenesis and progression of cancer, including cell proliferation, migration, invasion, metastasis, and apoptosis, by targeting PTEN, PDCD4, TIMP3, and RHOB [9–12] or by playing important roles in signaling pathways such as, RAS/MEK/ERK, PTEN/PI-3 K/AKT, and Wnt/β-catenin [13, 14]. Moreover, recent studies have shown that miR-21 is up-regulated in CRC [15, 16] and that high levels of tumoral miR-21 expression are associated with poor prognosis as well as poor response to chemotherapy in patients with CRC [17, 18].

Sec23A is one of two human Sec23 paralogs (Sec23A and Sec23B). Sec23A is a GTPase-activating protein, an integral component of the coat protein II complex that is critical for protein trafficking between the endoplasmic reticulum and Golgi apparatus [19, 20]. Emerging evidence suggests that Sec23A is involved in anti-tumorigenesis. A novel target for miR-375 and miR-200c, its expression is reduced in prostate cancer cells and tissues [21]. Moreover, as a direct target of miR-200s, Sec23A suppresses metastatic colonization and migration in breast cancer by mediating secretion of metastasis-suppressive proteins. Furthermore, *Sec23A* levels are significantly lower in clinical metastases relative to primary tumors [22].

Despite these findings of the biological roles of miR-21 and Sec23A, respectively in cancer, their relationship has not been established in CRC. Therefore, we aimed to investigate the functions of miR-21 and Sec23A as well as their relationship in CRC.

## Methods

### Cell lines and cell culture

CRC cell lines HT-29 (colorectal adenocarcinoma), SW-480 (Dukes’ type B), and DLD-1 (Dukes’ type C) representing different pathological stages of CRC were purchased from the Institute of Biochemistry and Cell Biology, Chinese Academy of Sciences (Shanghai, China). All cells were cultured in RPMI-1640 medium (Gibco, Carlsbad, CA) supplemented with 10 % fetal bovine serum (FBS; Bioind, Beit-Haemek, Israel) in a humidified 37 °C incubator supplemented with 5 % CO_2_.

### Plasmid transfection

Cells were transfected with pGCMV/EGFP plasmids containing hsa-miR-21 inhibitor or hsa-miR-21 mimic, or empty vector (negative control [NC]), or with pGPU6 plasmids containing Sec23A shRNA (sh-Sec23A), or control shRNA (sh-NC). The group which cells without treatment defined MOCK. All constructs were synthesized by GenePharma (Shanghai, China). SW-480 and DLD-1 cells were grown to 80–90 % confluence and then transfected. Transfection was carried out with Lipofectamine 2000 (Invitrogen, Shanghai, China; DNA/Lipofectamine 2000 ratio = 1/2.5) according to the manufacturer’s instructions. Six hours after transfection, the culture medium was replaced with fresh RPMI-1640 containing 10 % FBS. Stable transfectants were established by incubating cells in complete RPMI-1640 medium with Blasticidin (12 mg/mL; Sigma, Shanghai, China) for pGCMV/EGFP plasmids or G418 (500 mg/mL; Sigma) for pGPU6 plasmids for 15 days. Clones were verified by western blot and real-time quantitative polymerase chain reaction (RT-PCR), and the successful clones were pooled for the subsequent investigations.

### Cell proliferation assay

For the cell proliferation assays, SW-480 cells stably expressing miR-21 inhibitor, sh-Sec23A, or empty vector or control shRNA were seeded at a density of 2 × 10^3^ cells in 96-well plates and incubated for various periods of time (0 to 5 days). DLD-1 cells stably expressing miR-21 mimic, sh-Sec23A, or empty vector or control shRNA were seeded at a density of 1 × 10^3^ cells per well in 96-well plates and incubated for the same periods of time. Following incubation, Cell Counter Kit-8 (CCK-8; 10 μL) reagent was added to each well and cells were incubated at 37 °C for 1.5 h. Absorbance was measured at 450 nm using an electroluminescence immunosorbent assay reader as we described previously [23].

### Cell migration assay

SW-480 and DLD-1 cells were washed twice with serum-free RPMI-1640 medium and re-suspended in the same medium. Cells were seeded (SW-480, 1 × 10^5^; DLD-1, 1.5 × 10^5^) into the upper chambers of transwell culture plates, each with an 8-μm pore membrane insert (Corning, Shanghai, China). RPMI-1640 medium supplemented with 20 % FBS was placed in the lower chambers as a chemoattractant. After incubation for 48 h, cells that had penetrated through to the lower surface of the membrane were fixed with 4 % paraformaldehyde for 20 min, stained with crystal violet for 20 min at ambient temperature, photographed, and counted under a microscope (Nikon, Tokyo, Japan) at × 100 magnification in five randomly chosen fields.

### Cell invasion assay

The cell invasion assay was similar to the migration assay except that the transwell chambers were coated with matrigel solution (40 μL per chamber; matrigel:serum-free medium ratio 1:10). SW-480, (2 × 10^5^); or DLD-1, (1.5 × 10^5^) cells were seeded into the upper chambers of the transwells and RPMI-1640 medium with 20 % FBS (600 μL) was added to the lower chambers. After 48 h incubation, the cells that had penetrated the matrigel and moved to the lower surface of the membrane were fixed with 4 % paraformaldehyde and stained with crystal violet. Cells adhering to the upper surface of the membrane were removed with a cotton swab. The cells attached to the lower surface were counted and photographed under a microscope (Nikon) at × 100 magnification in five randomly chosen fields.

### Isolation of RNA and quantitative polymerase chain reaction analysis

Forty-eight hours after transfection or after tumor dissection, total RNA was extracted from cultured cells or ground tumor tissue using TRIzol (Invitrogen) according to the manufacturer’s protocols. Total miR-21 or Sec23A RNA (500 ng) was reverse transcribed to cDNA with miRNA-specific RT primers (RiboBio, Guangzhou, China) or random primers (TaKaRa, Dalian, China), respectively. Gene expression was measured by PCR using an Applied Biosystems 7500 Fast Sequence Detection System and SYBR Green PCR Kit (QIAGEN, Shanghai, China) under the following conditions: denaturation at 95 °C for 5 min, followed by 40 cycles of denaturation at 95 °C for 10 s and annealing and extension at 60 °C for 30 s. The relative miRNA and mRNA expression levels were normalized to U6 and β-actin expression, respectively.

### Western blot analysis

Seventy-two hours after transfection or tumor dissection, cells were harvested and subjected to lysis in the presence of a protease inhibitor cocktail and then to centrifugation at 14,000 *g* for 15 min at 4 °C. The supernatant fraction was collected and the protein concentration was measured by using a bicinchoninic acid protein assay kit (Beyotime, Hangzhou, China). An aliquot of 40 μg of denatured protein from each sample was treated with sodium dodecyl sulfate and applied to a 10 % polyacrylamide gel for electrophoretic separation, then transferred onto a nitrocellulose membrane. After blocking with 5 % nonfat milk for 2 h at ambient temperature, membranes were incubated with primary antibody (1:1000 dilution; Abcam, Shanghai, China) at 4 °C overnight and horseradish peroxidase-conjugated secondary antibody (1:1000 dilution, Abcam) for 1 h at ambient temperature. The blots were then incubated with enhanced chemiluminescence solution for 1 min. The signals were detected and quantified by densitometry using Quantity One software. GAPDH was used as an endogenous control.

### Tumor xenografts in mice

Fifteen male athymic BALB/c nude mice (4-week-old) were obtained from the Shanghai Medical Experimental Animal Care Commission (Shanghai, China). All animal procedures and experimental protocols were approved by Laboratory Animal Ethics Committee of Wenzhou Medical University. Based on in vitro findings, mice were randomized into 3 groups. The experiment was performed only once.

To establish xenograft tumors, DLD-1 cells (8 × 10^6^ in 200 μL of medium) stably expressing miR-21mimic were injected subcutaneously into the dorsal flank of each mouse. Other mice were injected with cells transfected with empty vector as NC as negative control or cells without treatment as MOCK. Each mouse’s tumor was measured weekly, beginning on day 7 after the injection, by a Vernier caliper along two perpendicular axes. The volume of the tumor was calculated with the formula: volume = (length × width^2^)/2. Twenty-one days after the injection, the mice were killed and the tumors were dissected for analyses.

### Immunohistochemical analysis

Tumor tissues were subjected to immunohistochemical analysis for Sec23A with a kit (Boster, Wuhan, China) used according to manufacturer’s instructions. Briefly, each tissue section was deparaffinized, rehydrated, and rinsed with phosphate-buffered saline solution (PBS). High- pressure antigen retrieval was carried out in citrate buffer, which was then removed by rinsing with PBS. The sections were incubated with 3 % H_2_O_2_ for 8 min and then with 5 % normal goat serum for 30 min. The sections were then sequentially incubated with specific primary antibody (anti-Sec23A, Abcam), biotinylated goat anti-rabbit IgG, and avidin-biotin-peroxidase complex and rinsed with PBS. The slides were stained with 3,3-diaminobenzidine, counterstained with hematoxylin, and photographed under a light microscope (×200 magnification).

### Statistical analyses

Data were analyzed with SPSS 17.0 software and are expressed as mean ± standard deviation (SD). Statistical significance of differences between groups was determined by analysis of variance (ANOVA) or two-tailed Student *t*-test. A *p-*value <0.05 was considered statistically significant. All experiments were performed at least three times.

## Results

### MiR-21 expression in CRC cells

**Fig. 1:**
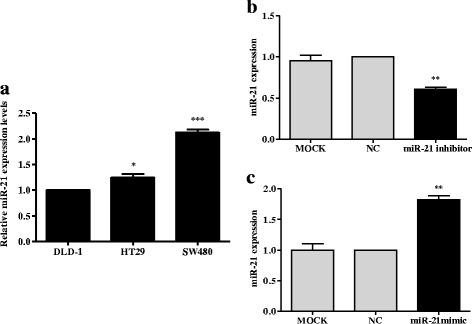
miR-21 is overexpressed in CRC cell lines. **a** Expression of miR-21 in untreated HT-29, SW-480, and DLD-1 cell lines. **b** The expression of miR-21 in SW-480 cells after transfection with miR-21 inhibitor, negative control (NC) or cells without treatment (MOCK). **c** The expression of miR-21 in DLD-1 cells after transfection with miR-21 mimic, negative control (NC) or cells without treatment (MOCK). * *p* < 0.05, ** *p* < 0.01, *** *p* < 0.001

Levels of miR-21 mRNA were significantly higher in HT-29 (colorectal adenocarcinoma) and SW-480 (Dukes’ type B) cells than in DLD-1 (Dukes’ type C) cells (*p* < 0.05, *p* < 0.001, respectively; Fig. 1a). The expression of miR-21 was significantly suppressed in SW-480 cells transfected with miR-21 inhibitor compared with negative controls (*p* < 0.01; Fig. 1b). In contrast, transfection of miR-21 mimic into DLD-1 cells significantly increased the expression of miR-21 compared with negative controls (*p* < 0.01; Fig. 1c).

### MiR-21 overexpression stimulates proliferation, migration, and invasion of CRC cells

**Fig. 2:**
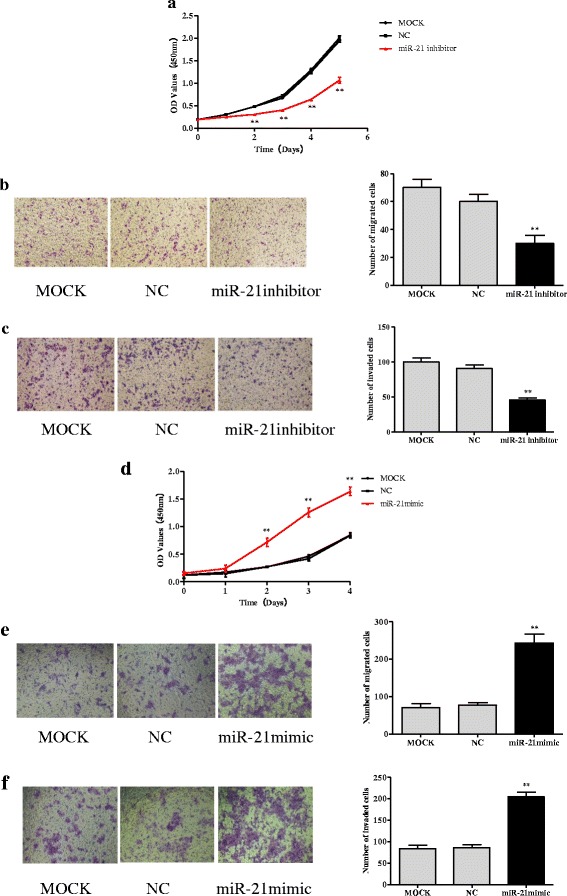
MiR-21 overexpression stimulates proliferation, migration, and invasion of CRC cells. **a**-**c** Inhibition of miR-21 in SW-480 by miR-21 inhibitor reduced cell proliferation, migration, and invasion relative to negative control (NC) and mock-treated cells (MOCK). **d**-**f** Up-regulation of miR-21 in DLD-1 by miR-21 mimic increased proliferation, migration, and invasion relative to negative control and mock-treated cells. In panels **b**, **c**, **e**, and **f**, photos on the *left* are representative images of migrated (**b**, **e**) and invaded cells on matrigel membranes (**c**, **f**), while the graphs on the *right* indicate quantification of cells. ** *p* < 0.01

Inhibition of miR-21 expression in SW-480 cells resulted in decreased proliferation, migration, and invasion compared with controls (all panels, *p* < 0.01; Fig. 2a-c). In contrast, cell proliferation, migration, and invasion were markedly increased in miR-21 over-expressing DLD-1 cells compared with the controls (all panels, *p* < 0.01; Fig. 2d-f).

### MiR-21 inhibits expression of Sec23A in CRC cell lines

**Fig. 3:**
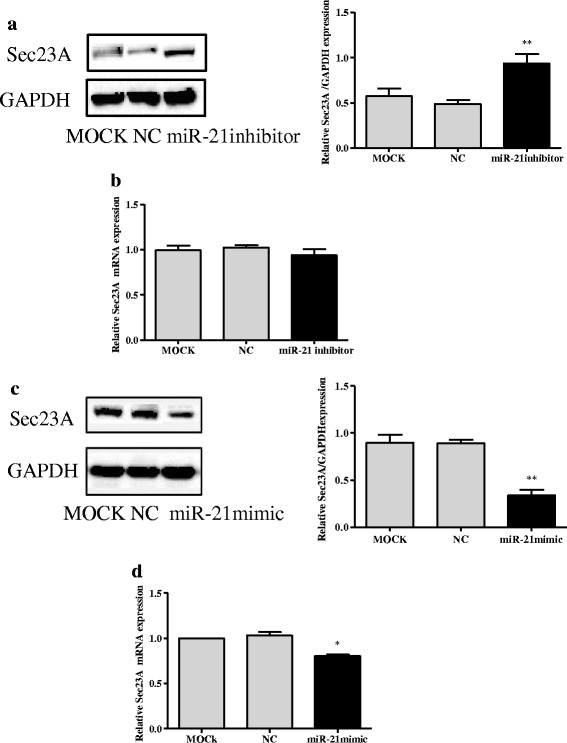
MiR-21 inhibits expression of Sec23A in CRC cell lines. **a** Inhibition of miR-21 expression promoted the expression of Sec23A protein in SW-480 cells relative to negative control (NC) and mock-treated cells (MOCK). **b** There was no significant difference in expression of Sec23A mRNA in SW-480 cells relative to negative control and mock-treated cells. **c**-**d** Over-expression of miR-21 by miR-21 mimic decreased the expression of Sec23A protein and mRNA in DLD-1 cells relative to negative control and mock-treated cells. Protein expression in these cells was determined by densitometric analysis. * *p* < 0.05, ** *p* < 0.01

Inhibition of miR-21 expression significantly increased the expression of Sec23A protein in SW-480 cells compared with controls (*p* < 0.01; Fig. 3a). There was no significant difference in expression of Sec23A mRNA between the SW-480 cells transfected with miR-21 inhibitor and controls (Fig. 3b). In contrast, miR-21 over-expression significantly suppressed the expression of Sec23A protein and mRNA in DLD-1 cells compared with controls (*p* < 0.01, *p* < 0.05, respectively; Fig. 3c-d).

### Expression of Sec23A and miR-21 in CRC cells

**Fig. 4:**
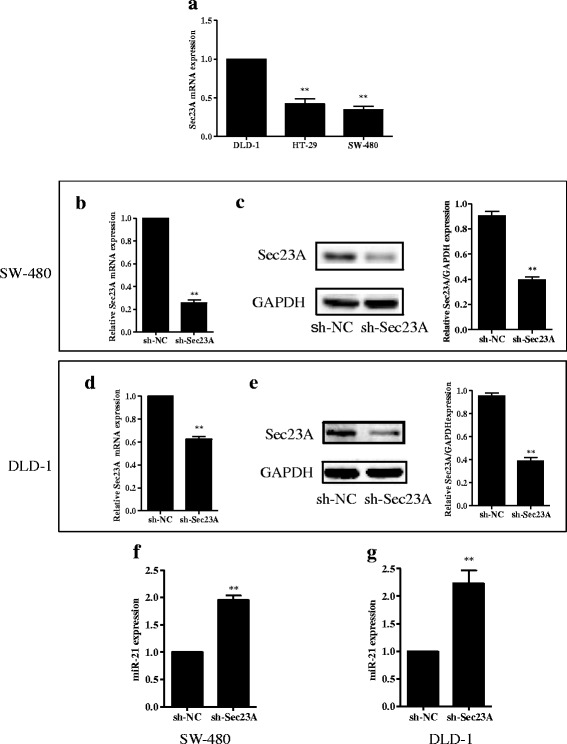
Sec23A knockdown increases expression of miR-21 in CRC cell lines. **a** Expression of Sec23A mRNA in untreated HT-29, SW-480, and DLD-1 cell lines. **b**-**c** Sec23A mRNA and protein expression was suppressed in Sec23A knockdown SW-480 cells. **d**-**e** Sec23A mRNA and protein expression was suppressed in Sec23A knockdown DLD-1 cells. Protein expression in these cells was quantified by densitometric analysis. **f**-**g** MiR-21 expression was increased in Sec23A knockdown SW-480 and DLD-1 cells. ** *p* < 0.01

Sec23A mRNA levels were lower in untreated HT-29 and SW-480 cells than in untreated DLD-1 cells (all *p* < 0.01; Fig. 4a). Sec23A mRNA levels did not differ significantly between HT-29 and SW-480 cells. The expression of Sec23A protein and mRNA levels in SW-480 or DLD-1 cells was significantly suppressed by transfection with sh-Sec23A compared with cells tranfected with sh-NC (all panels, *p* < 0.01; Fig. 4b-e). On the other hand, transfection with sh-Sec23A significantly increased the expression of miR-21 in SW-480 or DLD-1 cells compared with cells transfected with sh-NC (all *p* < 0.01; Fig. 4f-g).

### Downregulation of Sec23A promotes the proliferation, migration, and invasion of CRC cells

**Fig. 5:**
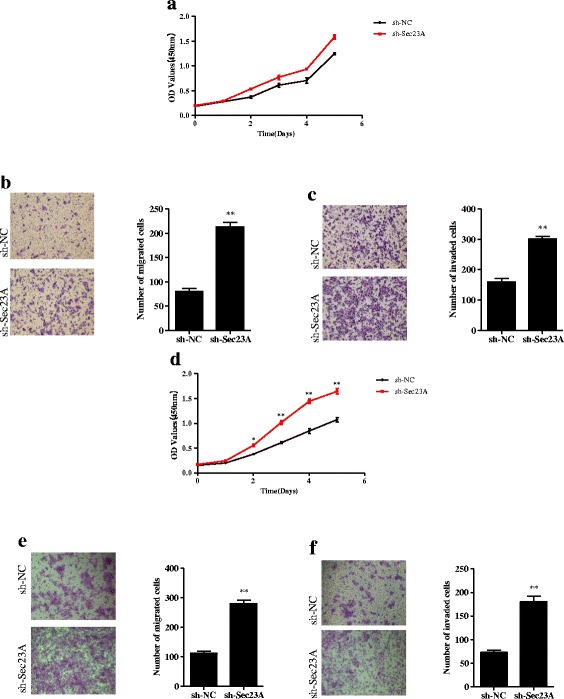
Downregulation of Sec23A promotes the proliferation, migration, and invasion of CRC cells. **a**-**c** Sec23A knockdown increased migration, and invasion of SW-480 cells relative to negative controls (sh-NC). **d**-**f** Sec23A knockdown increased proliferation, migration, and invasion of DLD-1 cells relative to negative controls. In panels **b**, **c**, **e**, and **f**, photos on the *left* are representative images of migrated (**b**, **e**) and invaded cells on Matrigel membranes (**c**, **f**) while the graphs on the *right* indicate quantification of cells. * *p* < 0.05, ** *p* < 0.01

Although downregulation of Sec23A had no significant effect on SW-480 cell proliferation, it did promote the migration and invasion of these cells compared with sh-NC transfectants (all *p* < 0.01; Fig. 5a-c). DLD-1 cells in which Sec23A expression was inhibited exhibited significantly greater proliferation, migration, and invasion (all *p* < 0.05–0.01) than cells transfected with sh-NC (Fig. 5d-f).

### MiR-21 overexpression promotes tumor growth in BALB/c nude mice

**Fig. 6:**
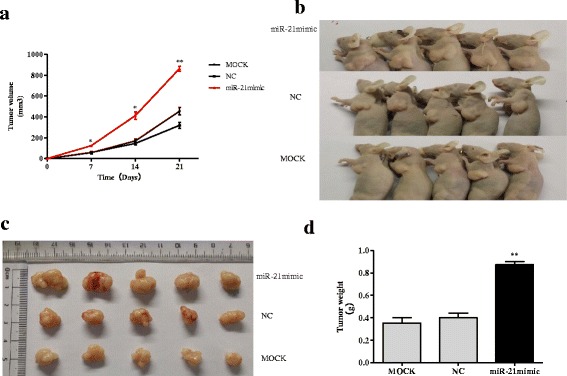
Over-expression of miR-21 enhances DLD-1 tumor growth in BALB/c nude mice. **a** Mice inoculated with miR-21 over-expressing DLD-1 cells grew larger tumors than mice inoculated with negative controls (NC) or mock-treated cells (MOCK). **b** Tumor-bearing mice before dissection. **c** The dissected tumors. **d** Tumors dissected from mice inoculated with miR-21 over-expressing DLD-1 cells weighed significantly more than tumors from mice inoculated with negative control or mock-treated cells. * *p* < 0.05, ** *p* < 0.01

**Fig. 7:**
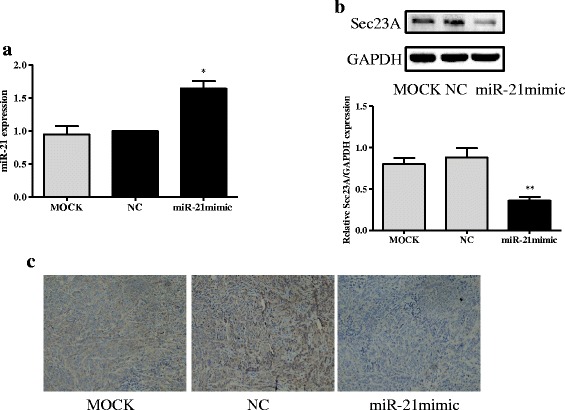
Greater expression of miR-21 in DLD-1 tumors is associated with lower levels of Sec23A protein expression. **a** Tumors from mice inoculated with miR-21 over-expressing DLD-1 cells expressed higher levels of miR-21 than tumors from mice inoculated with negative controls (NC) or mock-treated cells (MOCK). **b** Tumors from mice inoculated with miR-21 over-expressing DLD-1 cells expressed lower levels of Sec23A than tumors from mice inoculated with negative controls or mock-treated cells. **c** The expression of Sec23A in tumor tissues was determined by immunohistochemical staining; representative images are shown (original magnification, ×200). * *p* < 0.05, ** *p* < 0.01

Mice inoculated with miR-21 over expressing DLD-1 cells exhibited much faster tumor growth than the control or mock treatment groups (*p* < 0.01, *p* < 0.05, Fig. 6a). The weights of tumors dissected on day 21 from the mice inoculated with miR-21 over expressing DLD-1 cells were greater than those of the tumors from the control or mock group (*p* < 0.01; Fig. 6b-d). The expression of miR-21 was significantly higher in the tumors from the mice inoculated with miR-21 over expressing DLD-1 cells than in those from the control or mock treatment groups (*p* < 0.05; Fig. 7a). On the other hand, expression of Sec23A protein was significantly lower in tumors from the mice inoculated with miR-21 over expressing DLD-1 cells than in the tumors from the control or mock treatment groups (*p* < 0.01; Fig. 7b). This result was confirmed by immunohistochemical analysis showing lower Sec23A expression in the tumors from mice inoculated with miR-21 over expressing DLD-1 cells (Fig. 7c).

## Discussion

Our findings suggest that miR-21 promoted proliferation, migration, and invasion of SW-480 and DLD-1 CRC cells in vitro by down regulating the expression of Sec23A. Moreover, over-expression of miR-21 promoted tumor growth in BALB/c nude mice.

MiRNAs, dysregulated in many types of cancer, play important roles in tumorigenesis [24–26]. In light of previous studies indicating that miR-21 level is increased in CRC tissues and CRC cell lines [27, 28], we determined the effect of miR-21 on the tumorigentic activities of representative CRC cells as well as its relationship with Sec23A. The expression of miR-21 in SW-480 was higher than that in DLD-1 cells. There is no satisfactory explanation for this discrepancy because that DLD1 tends to be more metastatic than SW480 cells. The different effects on Sec23A protein and mRNA in SW-480 and DLD-1 cells were discovered in this study suggesting that the effects are cell line specific. Also, this may be attributed to indirect regulation of miR-21 and involvement of other regulators of the process of protein formation. Our data show that miR-21 was over expressed in HT-29 and SW-480 human CRC cells and that this over expression suppressed Sec23A expression in these cells. Up-regulation of miR-21 promoted CRC cell proliferation, migration, and invasion while down-regulation of miR-21 resulted in decreased proliferation, migration, and invasion of these cells. These findings are consistent with earlier reports that miR-21 induced invasion and metastasis [29] and that miR-21 over-expression enhanced CRC cell proliferation, migration, and invasion while miR-21 down regulation inhibited these cells’ proliferation, migration, and invasion [30–32]. Moreover, our finding that miR-21 over-expression promoted the growth of xenograft DLD-1 tumors in BALB/c nude mice is in line with a previous report that over-expression of miR-21 in colon cancer cells increased their tumorigenic potential in SCID mice [13].

In our study, downregulation of Sec23A stimulated the migration and invasion of SW-480 and DLD-1 cells and the proliferation of DLD-1, which is similar to the effects of miR-21 over-expression. This is consistent with the finding of Szczyrba et al. that inhibition of Sec23A in prostate carcinoma cells stimulated their proliferation [21]. In contrast, Korpal et al. suggested that Sec23A knockdown inhibits migration but promotes metastatic colonization of breast cancer cells [22]. These discoveries suggest that the effect of Sec23A knockdown may be different in different cell lines or stages of tumor. We also observed that knocking down the Sec23A significantly increases the expression of miR-21 in SW-480 and DLD-1 cells. This suggests a novel mechanism by which miR-21 contributes to tumorigenesis through downregulation of Sec23A. To identify the regulatory relationship between miR-21 and Sec23A, potential targets of miR-21 were analyzed by bioinformatics software, commonly used as targetscan for microRNA. Unfortunately, analysis showed that there were no binding sites between miR-21 and Sec23A. Thus, we couldn’t demonstrate that Sec23A is the direct target of miR-21, as verified by luciferase reporter assay [33, 34] of miR-21. There is probably a across talk between miR-21 and Sec23A but we cannot confirm for now how it works. The relationship and molecular pathways between miR-21 and Sec23A require further investigation. An experiment with double knock-in of miR-21 and Sec23A will be conducted in our future study to further demonstrate this. Also it would be interesting to determine the survival rate in each group in animal model and even to conduct clinical study to determine the association of miR-21 expression with clinic-pathological parameters in future study.

## Conclusion

MiR-21 is overexpressed in CRC cell lines and promotes proliferation, migration, and invasion in these cells in vitro associated with downregulation of Sec23A expression. Over-expression of miR-21 also promotes the growth of DLD-1 CRC tumors in BALB/c nude mice in vivo. These findings suggest that miR-21 might be a potential interesting target in CRC and may have therapeutic implications for patients with this disease.

## Abbreviations

FBS, fetal bovine serum; CRC, colorectal cancer; miR-21, microRNA-21; PBS, phosphate-buffered saline solution; RT-PCR, real-time quantitative polymerase chain reaction
